# Improving Clinical Care for Children with Congenital Hypothyroidism

**DOI:** 10.1097/pq9.0000000000000844

**Published:** 2025-09-11

**Authors:** Shadi Bakjaji, Kathryn Anglin, Trish Clotts, Bethany Dorsten, Kaitlyn Jones, Cindy Young, Samira Zoofan, Malak Abdel-Hadi, Cecilia Damilano, Kathryn Obrynba

**Affiliations:** * Division of Endocrinology, Department of Pediatrics, Nationwide Children’s Hospital, Columbus, Ohio; † The Ohio State University College of Medicine, Columbus, Ohio; ‡ Center for Clinical Excellence, Nationwide Children’s Hospital, Columbus, Ohio.

## Abstract

**Introduction:**

Congenital hypothyroidism (CH) is a common endocrine disorder that requires optimal management and intensive follow-up to prevent neurocognitive impairment, especially within the first 3 years of life.

**Methods:**

We implemented a quality improvement (QI) initiative to standardize care and reduce loss to follow-up for children younger than 3 years with CH. The project was conducted in a pediatric endocrinology clinic at a large tertiary hospital in the Midwestern United States from January 2021 to December 2024. The primary aim was to increase the percentage of patients younger than 3 years with CH who achieved 2 or more normal thyroid-stimulating hormone (TSH) levels within a rolling 12-month period, from a baseline of 77% to a goal of greater than 95%. A multidisciplinary QI team—endocrinologists, nurses, social workers, administrative staff, a certified QI specialist, and care coordinators—used Plan–Do–Study–Act cycles to optimize care. Data were collected monthly from the electronic medical record to identify patients who did not meet TSH targets or missed clinic visits. Key interventions included case management, standardized caregiver education, and identification of high-risk patients. The monthly cohort consisted of 74–124 children younger than 3 years with CH.

**Results:**

After 12 months of intervention, this initiative increased the percentage of unique patients younger than 3 years with CH who achieved 2 normal TSH values within a rolling 12-month period, from a baseline of 77%–94%—an improvement that has been sustained.

**Conclusions:**

This QI initiative highlights the importance of interdisciplinary collaboration in improving the clinical care of pediatric patients with CH.

## INTRODUCTION

Congenital hypothyroidism (CH) is a common and serious endocrine disorder, affecting approximately 1 in every 2,000 to 4,000 live births worldwide.^1^ Left untreated, CH can lead to severe intellectual disabilities, impaired growth, and developmental delays, especially during the critical first few years of life when brain development is most sensitive to thyroid hormone levels.^2–7^ Early identification and optimal treatment of CH are crucial for preventing these adverse outcomes. Since the introduction of newborn screening programs in the 1970s, the early detection of CH has significantly improved, allowing for timely intervention with levothyroxine (L-T4) therapy.^4–8^ However, despite these advancements, gaps remain in the ongoing management of CH, particularly in the area of consistent clinical and laboratory follow-up to ensure optimal treatment outcomes.^9,10^

The American Academy of Pediatrics (AAP) and the European Society of Pediatric Endocrinology have established clear guidelines for the management of CH. Additionally, the Pediatric Endocrine Society provides peer-reviewed referral guidance to assist clinicians in evaluating and referring children with abnormal newborn screens for CH.^11^ These guidelines emphasize the importance of regularly monitoring thyroid function through clinical evaluations and laboratory testing during the first 3 years of life.^12,13^ Specifically, thyroid-stimulating hormone (TSH) and free thyroxine levels should be assessed every 1–2 weeks after the initiation of therapy until the serum TSH level is normal, and then at progressively longer intervals throughout infancy and early childhood.^13^ This intensive follow-up aims to ensure that thyroid hormone levels remain within the therapeutic range, thus avoiding the risk of suboptimal treatment, which could lead to cognitive deficits and developmental delays.^14,15^

Inadequate treatment or loss of follow-up can have serious consequences for children with CH. In some cases, prolonged periods without proper thyroid hormone replacement can result in irreversible intellectual impairment.^2,16^ As a result, the continuous and precise management of thyroid hormone levels is critical for these patients. Nevertheless, a significant portion of children with CH are lost to follow-up or fail to achieve consistent normal TSH values due to various barriers, including healthcare system challenges, patient nonadherence, or issues with clinical workflows.^17^

In this context, we initiated our quality improvement (QI) project to address a gap in the clinical management of CH in a pediatric endocrinology clinic at a large tertiary care center. The project focused on increasing the percentage of unique patients younger than 3 years with CH who achieve at least 2 normal TSH values within a 12-month rolling period. Before the intervention, the baseline percentage of patients meeting this criterion was 77%. We aimed to increase this percentage to a high-reaching goal of 95% through targeted interventions designed to enhance both clinical and laboratory follow-up.

## METHODS

### Setting and Improvement Team

**Fig. 1.**
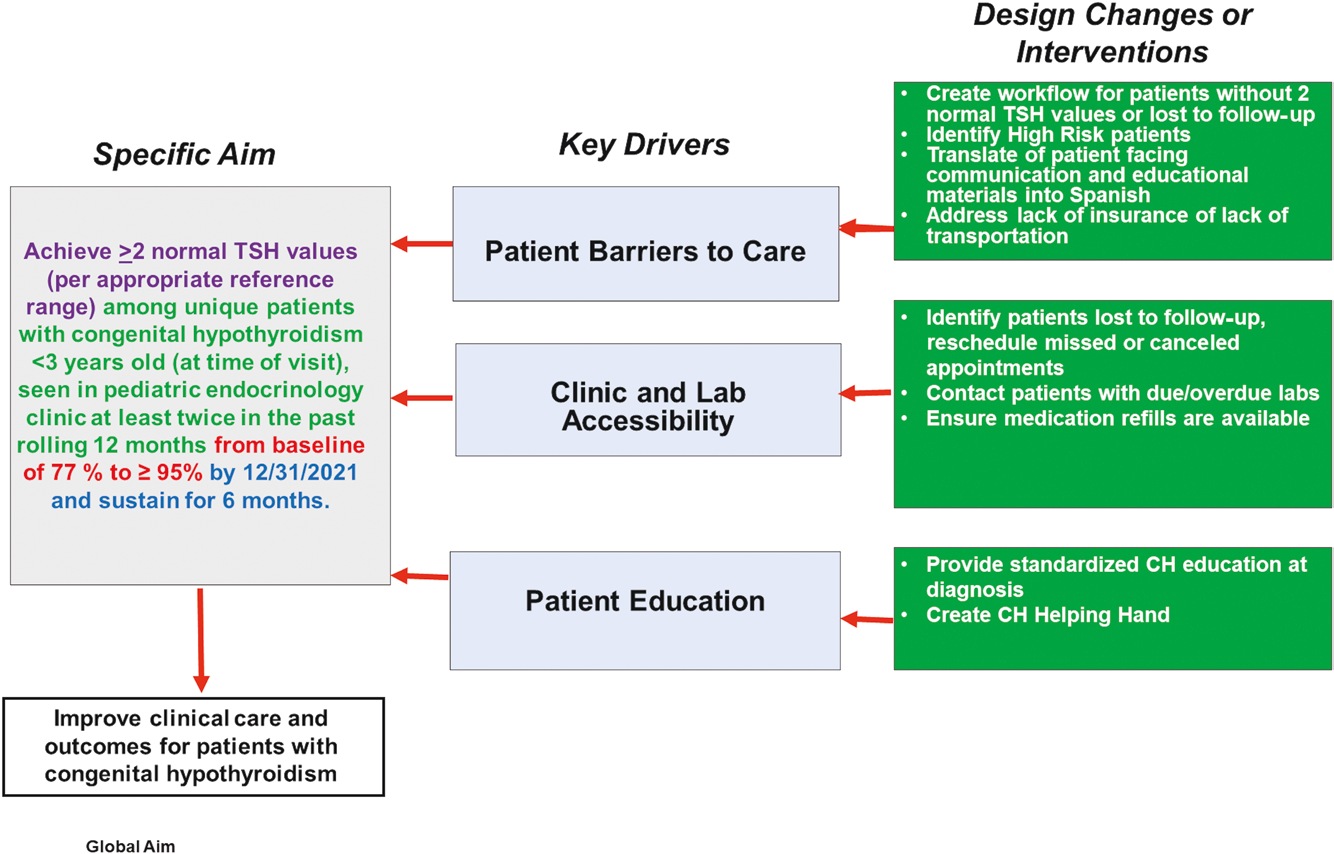
Key driver diagram for the QI initiative to optimize management and prevent loss to follow-up in children younger than 3 years old with congenital hypothyroidism.

A QI initiative was developed within a pediatric endocrinology clinic at a large tertiary academic pediatric hospital located in the Midwest, United States, to improve and standardize care and prevent loss to follow-up for children younger than 3 years diagnosed with CH. The primary aim was to increase the percentage of patients younger than 3 years with CH who achieved at least 2 normal TSH values within a rolling 12-month period. The multidisciplinary QI team consisted of endocrinology physicians, fellows, nurses, social workers, a certified QI leader/specialist, administrative assistants, and care coordinators. Our pediatric endocrinology clinic sees approximately 8,000 patient visits annually, including 2,700 unique patients. Children with CH are followed as part of this broader population. Patients are evaluated and managed by a team of 15 board-certified pediatric endocrinologists and 3 nurse practitioners. CH management is guided by the AAP recommendations, which advise monitoring TSH and free T4 every 1–2 months during the first 6 months of life, every 2–3 months during the second 6 months, and every 3–4 months from 1 to 3 years of age.^13^ To clarify the project’s objectives and guide the team’s efforts, a key driver diagram was developed using QI and Lean Six Sigma tools (Fig. 1). The interventions were implemented from January 2021 to December 2024.

### Interventions

#### Case Management and Process Tracking

The QI team conducted monthly meetings to review the case management of target patients and identify gaps in patient care needs. The primary outcome measure was the percentage of children younger than 3 years with CH who achieved at least 2 normal TSH values within a rolling 12-month period. In addition, process measures were tracked monthly using electronic reports from the electronic medical record (EMR). These included the number of patients with missed or canceled endocrinology appointments, overdue laboratory work, and other gaps in follow-up. These process measures helped identify patients needing case management interventions to achieve the outcome goal. Patients were identified monthly using International Classification of Diseases, 10th Revision codes through EMR queries. These data were reviewed during team meetings to assess follow-up, laboratory completion, and progress toward TSH targets. Some laboratories were completed at outside facilities; results were either imported via health information exchange or manually retrieved and entered into the EMR.

The number of eligible patients ranged from 74 to 124 per month, with 92 at project launch. Each month, 15–30 patients were flagged for follow-up due to abnormal laboratory results or missed appointments. This number declined over time as more patients met goals. Variability reflected the dynamic nature of the cohort, as children aged out and new diagnoses were added. The population size allowed manual monthly review of all eligible patients, regardless of visit timing.

We contacted flagged families by phone or EMR messaging. If contact was unsuccessful, the patient remained on the list for monthly review. Outreach continued until the issue (eg, missed laboratory or visit) was resolved.

Case management for individual patients was divided among physician teams, and their respective nurses and administrative assistants, to clearly identify responsibilities for follow-up action. Endocrinology nurses contacted families via patient portal messaging, phone, or letter to remind them of due or overdue laboratory tests and ensured that medication refills were available. Administrative assistants scheduled follow-up appointments for those who were “to be seen” and for those who had been identified as having missed or canceled their clinic visits. Social workers and care coordinators addressed patient-specific concerns such as lack of insurance or transportation, which could otherwise delay care. These case reviews enabled the QI team to identify and address care gaps effectively, as well as efficiently identify any additional barriers to care using a team-based approach, and personalize interventions for each patient. Plan–Do–Study–Act (PDSA) cycles were used throughout the initiative to continuously refine and optimize this workflow.

#### Standardized Caregiver Education

At the time of diagnosis, endocrinology nurses provided families with standardized education on CH and the correct administration of L-T4. As part of this education, patient caregivers received a Congenital Hypothyroid Helping Hand, a standardized resource summarizing the diagnosis, treatment, and management of CH in infants and children. (**See Supplemental Digital Content 1**, which displays the Congenital Hypothyroid Helping Hand educational resource used in this QI initiative. It is available online at Nationwide Children’s Hospital, https://links.lww.com/PQ9/A702.) Helping Hand instructions are intended as a supplement to verbal instructions provided by medical professionals and are developed by medical, nursing, and allied health professionals at our institution. The information on the Congenital Hypothyroid Helping Hand was reviewed and revised by the QI team to reflect our current practice and standard of care. This material was initially written in English and translated into Spanish to reduce health disparities in non–English-speaking families. Education delivery was documented in the EMR and tracked by nurses at diagnosis. Minor early gaps were resolved as this became a routine part of care.

#### Identification of High-risk Patients

High-risk patients were identified as those who consistently failed to achieve 2 normal TSH values within a rolling 12-month period, frequently missed scheduled appointments or laboratory follow-ups, faced accessibility challenges, were followed by Child Protection Services, or belonged to families with literacy or emotional difficulties. These high-risk patients, regardless of their most recent TSH values, were tracked monthly by the QI team to ensure comprehensive and consistent management. On average, 5–10 patients were tracked as high-risk per month, a number that remained relatively stable throughout the project. These patients were discussed at every case management meeting, and interventions were tailored to address specific barriers to care. In some cases, additional follow-up calls, social work outreach, or closer visit intervals were arranged. If families could not be reached after 3 outreach attempts, our social worker mailed a certified letter to the home address on file, advising of the need for urgent follow-up and offering support to reengage with care.

## RESULTS

**Fig. 2.**
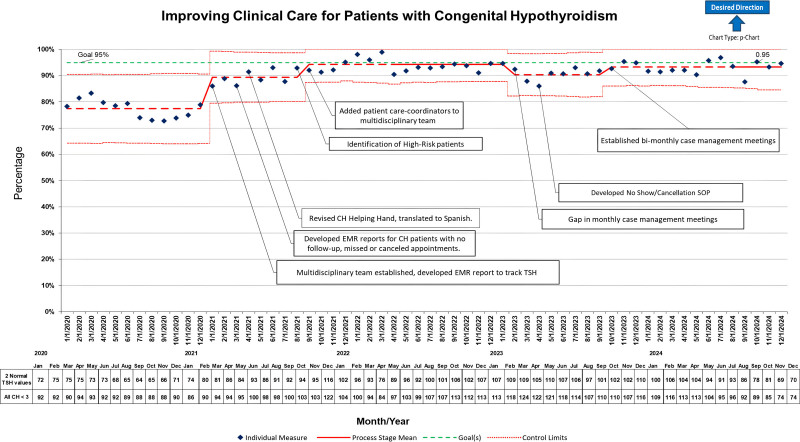
Percentage of children younger than 3 years old with congenital hypothyroidism achieving 2 or more normal TSH values in a rolling 12-month period, before and after implementation of the QI initiative. SOP indicates standard operating procedure.

This QI initiative increased the percentage of patients younger than 3 years with CH who achieved at least 2 normal TSH values within a rolling 12-month period, from our baseline of 77%–94% after 12 months of interventions. As shown in Figure 2, the percentage of patients meeting the TSH target increased progressively after implementation of the QI initiative, rising from a baseline of 77%–94%, and remained above preintervention levels over time. Figure 2 reflects both pre- and postintervention trends within a single control chart. Consistent improvement in performance was recognized within the first 8 months of project initiation and intervention, with a centerline shift in the baseline from 77% to 89%. This improvement was followed by further consistent improvement, reaching 94% performance by 12 months of intervention. This high level of performance was maintained for 13 months, well beyond the initial project period. Despite this project not meeting the initial aspirational goal of exceeding 95% performance within the first 12 months, we have achieved the 95% target within individual months every year since project initiation. As shown in Figure 2, the project’s performance has consistently been in the 90%–95% range.

During a temporary gap in our regular monthly meetings, we observed a temporary performance decline to 90%. This decline coincided with several identified factors, including temporary staffing shortages and staff transitions. The QI team implemented other interventions as outlined in Figure 2 to help maintain and sustain high performance results.

## DISCUSSION

This QI project improved the clinical care of young children with CH by optimizing their clinical management and reducing the risk of loss to follow-up. Although we cannot conclude that loss to follow-up was entirely prevented, our interventions—such as regular case tracking and immediate rescheduling of missed visits—contributed to improved care continuity. Our initiatives led to a substantial increase in the percentage of patients younger than 3 years with CH who achieved 2 or more normal TSH levels within a rolling 12-month period. We accomplished this by leveraging the EMR to identify target patients for case management by the QI team, providing focused education for caregivers and families, and by identifying high-risk patients for appropriate allocation of resources. This improvement effort demonstrates that by using QI methodology and a team-based approach, identification and mitigation of barriers can optimize care.

To assess the potential impact of the COVID-19 pandemic on our outcomes, we reviewed 2019 data, which showed that 69 of 78 (88%) patients younger than age 3 years with CH met the TSH target—higher than the 77% observed in 2020, the year used as our baseline. Although 2020 marked the onset of the pandemic, in-person endocrine visits were suspended for approximately 2 months before transitioning to telehealth with continued laboratory monitoring. However, we recognize that care delivery disruptions during this period may have affected appointment adherence and laboratory completion. Although this introduces a limitation in using 2020 as a baseline, postintervention rates not only rebounded but also surpassed prepandemic levels and remained consistently high, suggesting the observed improvement was not solely due to the resumption of standard care, but likely reflects the sustained impact of our structured QI interventions.

The AAP guidelines recommend intense follow-up during the first 3 years of life, but implementation of these guidelines can be challenging in busy clinical settings, leading to missed appointments or delays in laboratory evaluations. By focusing on improving the consistency of follow-up care, we sought to ensure that children with CH in our clinic received optimal thyroid hormone replacement, thereby minimizing the risk of intellectual disability and other developmental issues.

Our QI project aligns with existing literature on the importance of timely diagnosis and treatment of CH. Numerous studies have shown that early and adequate L-T4 therapy, initiated within the first few weeks of life, is associated with grossly normal neurocognitive outcomes.^13–16,18^ However, even when treatment is initiated on time, ongoing management must be meticulous in maintaining thyroid function within the desired range. Gaps in care, particularly in the monitoring phase, have been linked to poorer cognitive outcomes, including deficits in memory, visuospatial processing, and sensorimotor function.^18,19^

In response to these challenges, we implemented several strategies aimed at improving clinical care, optimizing thyroid hormone management, and follow-up adherence in our patient population. These strategies included more robust laboratory results and appointment tracking, as well as educational efforts to engage families in the importance of optimal CH care, and enhanced communication between families and care teams. By addressing these key areas, we aimed to close the care gap and ensure that children with CH in our clinic were receiving the high-quality, evidence-based care recommended by international guidelines. We believe these results and learnings from this initiative can potentially be applied to other settings and contribute to broader efforts to reduce the global burden of CH-related intellectual disability.

## IMPLICATIONS FOR CLINICAL PRACTICE

Early and consistent thyroid hormone regulation is critical for neurodevelopment in children with CH. Our intervention ensured that more children achieved target TSH levels on time. The use of a key driver diagram (Fig. 1) and continuous data-driven monitoring (Fig. 2) proved crucial in maintaining focus on this outcome.

Moreover, the success of this initiative illustrates the importance of regular tracking and follow-up, especially for high-risk patients, to prevent gaps in care. Incorporating EMR data in identifying and flagging patients who require additional attention helped the team intervene before any delays in care could arise. This type of system-based approach could serve as a model for other clinics seeking to improve outcomes in pediatric endocrinology or other chronic conditions that also require close laboratory monitoring.

## SUSTAINABILITY AND FUTURE DIRECTIONS

To build on our success, future PDSA cycles may explore enhanced patient education tailored to different cultural and linguistic backgrounds, addressing ongoing health disparities. Although we translated our educational materials into Spanish, expanding resources for other non–English-speaking populations could further reduce barriers to optimal care.

To our knowledge, few QI initiatives have addressed loss to follow-up in children with CH. In comparison to prior work, our QI initiative shares similarities with the project described by Matlock et al,^16^ which also aimed to reduce loss to follow-up in children younger than 3 years with CH. Both projects used EMR-based patient identification and multidisciplinary teams to implement structured interventions. However, our approach differed in several important ways. Although Matlock et al^16^ defined loss to follow-up based on time since the last visit, we incorporated a biochemical criterion—lack of 2 normal TSH values within a rolling 12-month period—providing a more dynamic and clinically relevant marker. Furthermore, our project emphasized proactive, real-time tracking of at-risk patients rather than retrospective outreach and included standardized, multilingual patient education materials to promote health equity. The integration of care coordinators and administrative staff in our bimonthly review process also reflects a more comprehensive team-based strategy. These distinctions highlight our project’s contribution to the field by expanding on existing QI frameworks to further optimize early CH management and follow-up.

## LIMITATIONS

Although the initiative successfully improved outcomes within our target group, the project has several limitations. First, the study was conducted within a single institution, which may limit the generalizability of our results. Different healthcare systems with varied patient demographics and resources may experience different outcomes when implementing similar strategies. Additionally, although we were able to maintain performance during the study period, long-term follow-up is necessary to determine whether this level of achievement can be sustained indefinitely, particularly as patients age and their care transitions. Finally, the reliance on EMR for patient identification and tracking, although efficient, may have led to occasional gaps in data entry or missed appointments that were not adequately captured.

Future initiatives should consider integrating best practice alerts within the EMR, similar to those used in other conditions, such as elevated blood pressure in patients with type 1 diabetes.^20^ These alerts could notify providers when a child younger than 3 years old with CH has not had normal thyroid function tests within the previous 4 months, or when a follow-up appointment with endocrinology has not been scheduled. This could further enhance patient tracking and care coordination. Future PDSA cycles should consider including caregiver burden from increased contact or testing to ensure a more holistic approach to care delivery.

## CONCLUSIONS

This QI initiative demonstrates the value of a structured, multidisciplinary approach to improving the care of young children with CH. By achieving and sustaining a high percentage of patients with normalized TSH values, we have shown that system-based interventions and ongoing collaboration among care providers can lead to meaningful improvements in patient outcomes.

## ACKNOWLEDGMENTS

The authors express their gratitude to the children and families who participated in this QI initiative. Special thanks to the QI leadership and the entire multidisciplinary team at Nationwide Children’s Hospital for their commitment to improving the care for children with CH.

## Supplementary Material


