# Supplementary material for: Improving Clinical Care for Children with Congenital Hypothyroidism
Source: Pediatr Qual Saf. 2025 Sep 11;10(5):e844. doi: 10.1097/pq9.0000000000000844 (PMC12425089; doi:10.1097/pq9.0000000000000844)
Supplement: Supplementary file 1 [file pqs-10-e844-s001.pdf]

Helping Hand™ *Health Education for Patients and Families*

Hipotiroidismo congénito
Congenital Hypothyroidism (Spanish)

Hipotiroidismo significa que el cuerpo no produce suficiente hormona tiroidea para el crecimiento normal, el desarrollo óseo y para controlar la forma en que el cuerpo utiliza la energía para funcionar (metabolismo). Cuando el metabolismo se ralentiza, muchas funciones corporales se ralentizan.

A los recién nacidos se les hacen pruebas para detectar el hipotiroidismo al nacer. Se habla de hipotiroidismo congénito cuando un bebé nace sin suficiente hormona tiroidea. El tratamiento de los niveles bajos de tiroides debe iniciarse en las primeras semanas de vida para evitar daños permanentes en el cerebro y problemas de aprendizaje.

Señales y síntomas

La mayoría de los bebés no presentan síntomas de niveles bajos de tiroides al nacer. Los síntomas que pueden desarrollarse poco después del nacimiento son:

- Color amarillo (ictericia) de la piel o los ojos
- Tono muscular flojo o el cuerpo parece flácido
- Un bulto o protuberancia alrededor del ombligo (hernia)
- Problemas de alimentación
- Estreñimiento
- Lengua agrandada

Tratamiento

El tratamiento del hipotiroidismo es sencillo, seguro y eficaz.
Le dará a su bebé una pastilla cada día para reponer la hormona tiroidea que le falta. El medicamento se llama levotiroxina (Synthroid®).

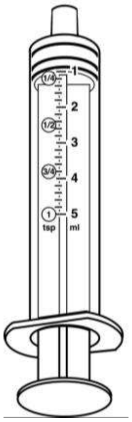

Imagen 1 Jeringa sin aguja para medicamentos

Como los bebés no pueden tragar una píldora, tendrá que:

1. Triturar la píldora.

©2012, revisado en 2023, Nationwide Children's Hospital
Continuación...

2. Mezclarla con 0.2 ml de agua estéril, leche humana o fórmula.
3. Dársela a su bebé con una jeringa sin aguja o una cuchara (Imagen 1). El médico o el profesional de atención médica de su hijo le indicará cuál debe utilizar.

No ponga el medicamento en el biberón de su bebé. Si no se termina el biberón, no recibirá suficiente medicamento.

Consultas de seguimiento

Su bebé visitará a su médico o profesional de atención médica cada 2 o 3 meses. Se le hará un análisis de sangre en cada visita para asegurarse de que está recibiendo la dosis correcta de medicamento. Puede que sea necesario analizarla más a menudo. Es probable que su bebé necesite medicamentos para la tiroides y análisis de sangre regulares durante el resto de su vida.
